# A virus-like particle-based tetravalent vaccine for hand, foot, and mouth disease elicits broad and balanced protective immunity

**DOI:** 10.1038/s41426-018-0094-1

**Published:** 2018-05-18

**Authors:** Wei Zhang, Wenlong Dai, Chao Zhang, Yu Zhou, Pei Xiong, Shuxia Wang, Xiaohua Ye, Qingwei Liu, Dongming Zhou, Zhong Huang

**Affiliations:** Vaccine Research Center, CAS Key Laboratory of Molecular Virology & Immunology, Institut Pasteur of Shanghai, Chinese Academy of Sciences, University of Chinese Academy of Sciences, Shanghai, 200031 China

## Abstract

Hand, foot, and mouth disease (HFMD) is an infectious disease that mainly affects infants and children, causing considerable morbidity and mortality worldwide. HFMD is commonly caused by enterovirus 71 (EV71) and coxsackieviruses A16 (CVA16), A6 (CVA6), and A10 (CVA10). Formalin-inactivated EV71 vaccines are currently available in China; however, these vaccines fail to confer cross-protection against infections by other HFMD-causing enteroviruses, highlighting the necessity of developing a multivalent HFMD vaccine. Our previous studies demonstrated that recombinant virus-like particles (VLP) of EV71, CVA16, and CVA6 are capable of inducing protective immunity against homologous virus challenges in mice. In this study, we generated CVA10-VLP using a baculovirus-insect cell expression system and then combined CVA10-VLP with EV71-VLP, CVA16-VLP, and CVA6-VLP to formulate a tetravalent VLP vaccine. Immunogenicity and protective efficacy of tetravalent VLP vaccine was compared with that of monovalent VLP vaccines. Mouse immunization studies revealed that the tetravalent vaccine elicited antigen-specific and long-lasting serum antibody responses comparable to those elicited by its corresponding monovalent vaccines. Moreover, tetravalent vaccine immune sera strongly neutralized EV71, CVA16, CVA10, and CVA6 strains with neutralization titers similar to those of their monovalent counterparts, indicating a good compatibility among the four antigens in the combination vaccine. Importantly, passively transferred tetravalent vaccine-immunized sera conferred efficient protection against single or mixed infections with EV71, CVA16, CVA10, and CVA6 viruses in mice, whereas the monovalent vaccines could only protect mice against homotypic virus infections but not heterotypic challenges. These results demonstrate that the tetravalent VLP vaccine represents a promising broad-spectrum HFMD vaccine candidate.

## Introduction

Hand, foot, and mouth disease (HFMD) is a highly contagious viral disease worldwide, especially in the Asia-Pacific region, and has led to significant morbidity and mortality^1,2^. The disease mostly affects infants and young children but occasionally occurs in older kids and adults^1^. HFMD is generally a mild and self-limiting disease characterized by fever, rashes on the hands and feet, and mouth sores. In some cases, however, the patients may develop serious neurological and cardiopulmonary complications that may result in fatal outcomes^1–3^. Historically, HFMD was commonly caused by enterovirus 71 (EV71) and coxsackievirus A16 (CVA16);^3–5^ however, in recent years, a large number of HFMD cases were found to be associated with coxsackievirus A6 (CVA6) and coxsackievirus A10 (CVA10) infections^6,7^. Moreover, CVA6 and/or CVA10 have been responsible for the recent and numerous HFMD outbreaks in many countries, such as Finland^8^, France^9,10^, Singapore^11^, Japan^12,13^, Spain^14^, Thailand^15^, and China^16,17^. Therefore, CVA6 and CVA10 have emerged as two major causative agents of HFMD. Furthermore, recent epidemiological surveys show that CVA6, CVA10, CVA16, and/or EV71 can co-circulate^8,9,11^, possibly leading to viral co-infections and genetic recombination, making it more difficult to control HFMD. In addition, EV71 infections have been more commonly associated with severe HFMD^18,19^, but infections with CVA16, CVA10, or CVA6 can also result in serious complications and even death^7,20–22^.

Currently, no approved antiviral therapy is available for HFMD. Vaccination has been considered as the most effective strategy to control and prevent this disease. Previous HFMD vaccine studies were mainly focused on developing EV71 vaccines^23,24^. To date, three formalin-inactivated EV71 whole-virus vaccines have been approved for human use and are commercially available in China^25^. However, these EV71 vaccines cannot provide effective protection against other major causative agents of HFMD, such as CVA16, CVA10, and CVA6^24^. Several experimental vaccines have been developed for CVA16, CVA10, and CVA6^26–29^, but no cross-protection was observed among these different enteroviral serotypes^24^. Therefore, to offer more comprehensive protection for HFMD, it is necessary to develop multivalent vaccines containing EV71, CVA16, CVA6, and CVA10 antigens^30,31^.

Recombinant virus-like particles (VLPs) are considered a very attractive and potent platform for viral vaccine development because of their high immunogenicity and safety; two good examples are the successful commercialization of VLP-based hepatitis B virus and human papillomavirus vaccines^32,33^. Previously, our group generated separate EV71-VLP, CVA16-VLP, and CVA6-VLP by employing a baculovirus-insect cell expression system and further demonstrated that these VLPs exhibit good immunogenicity and protective effects in their respective mouse models^27,29,34^. In the present study, we attempted to produce CVA10-VLP using the same strategy and then combined EV71-VLP, CVA16-VLP, CVA6-VLP, and CVA10-VLP together to construct a tetravalent VLP vaccine and tested its protective efficacy in mice. Our results showed that the tetravalent VLP vaccine can confer efficient and broad protection against EV71, CVA16, CVA10, and CVA6 viral infections, thus representing a promising broad-spectrum HFMD vaccine candidate.

## Results

### Expression and characterization of CVA10-VLP

**Fig. 1:**
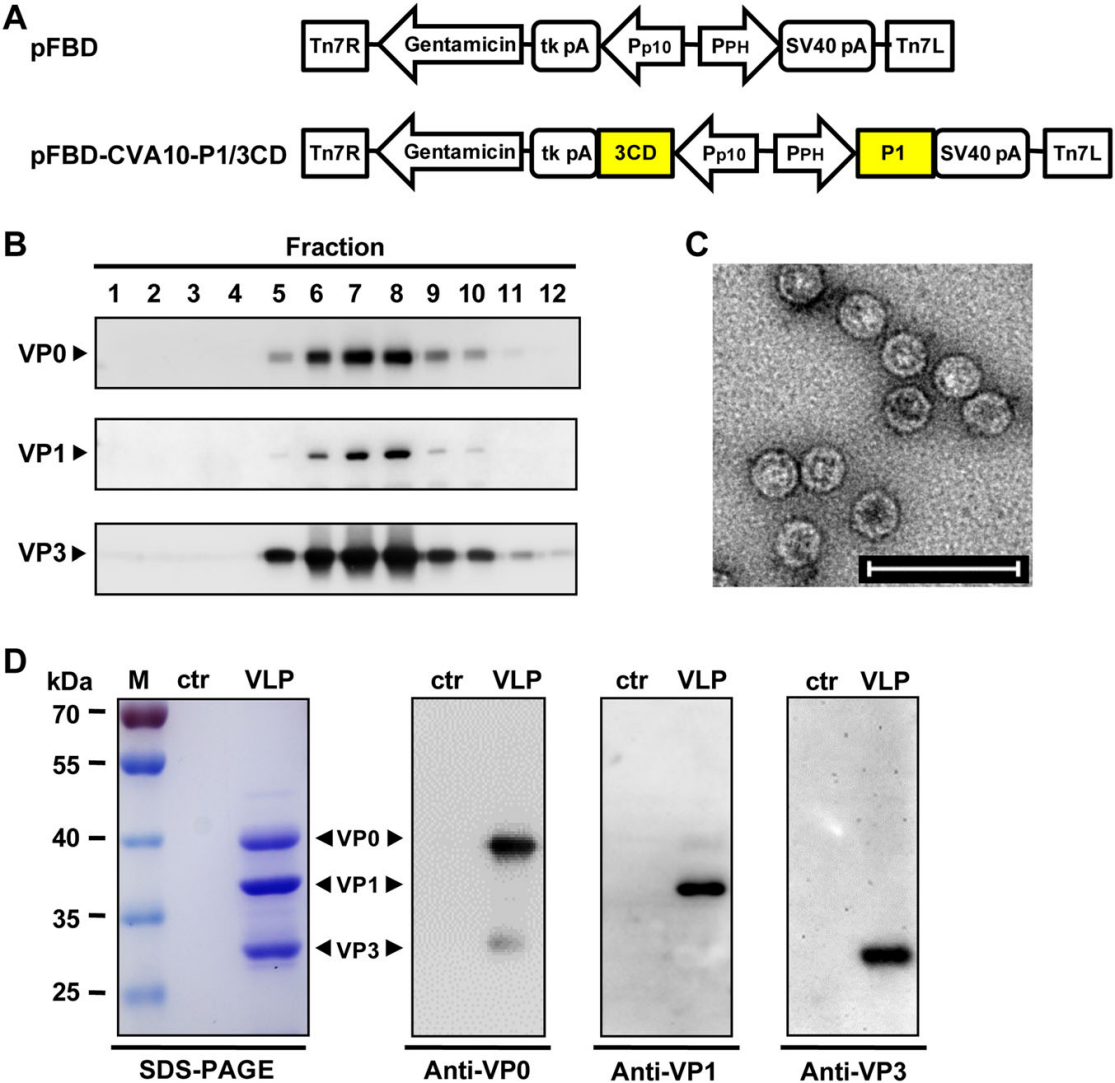
Expression and characterization of CVA10-VLP in insect cells. **a** Diagrams of plasmids pFBD and pFBD-CVA10-P1/3CD. Tn7R and Tn7L, right and left elements of Tn7 transposon; Gentamicin, gentamicin resistance gene; tk pA, herpes simplex virus thymidine kinase (tk) polyadenylation signal; P_p10_, *Autographa californica* multiple nuclear polyhedrosis virus (AcMNPV) p10 promoter; P_PH_, AcMNPV polyhedrin promoter; SV40 pA, SV40 polyadenylation signal. **b** Sucrose gradient sedimentation analysis. Lysates from Sf9 cells infected with baculovirus Bac-CVA10-P1/3CD were subjected to 10–50% sucrose gradient centrifugation. Twelve fractions were collected from the top of the gradient, followed by western blotting analysis using polyclonal antibodies against CVA10 VP0, VP1, and VP3 proteins. **c** Transmission electron microscopy imaging of CVA10-VLP. Scale bar = 100 nm. **d** SDS-PAGE and western blotting analysis of the purified CVA10-VLP sample. Lane M, protein marker; ctr, control antigen produced from uninfected Sf9 cells; and VLP, purified CVA10-VLP

It has been reported that the simultaneous expression of P1 precursor proteins and 3CD proteases of EV71, CVA16, or CVA6 in insect cells leads to the cleavage of P1 by 3CD into three capsid subunit proteins, namely, VP0, VP1 and VP3, all of which can spontaneously assemble into VLPs^27,29,35^. In the present study, the same expression strategy was used to prepare CVA10-VLP. The CVA10 P1 and 3CD gene fragments were separately cloned into the pFastBac™ Dual vector (pFBD) under the control of the polyhedrin (PH) and p10 promoters, respectively, to generate the plasmid, pFBD-CVA10-P1/3CD (Fig. 1a). This plasmid was used to produce the recombinant baculovirus, Bac-CVA10-P1/3CD, using the Bac-to-Bac baculovirus expression system. *Spodoptera frugiperda* Sf9 insect cells were infected with Bac-CVA10-P1/3CD, and the cell lysates were harvested at 3 days post-infection (dpi) and then subjected to sucrose gradient ultracentrifugation. The resultant fractions were tested for expression and distribution of CVA10 proteins by western blotting. As shown in Figure 1b, proteins were detected at the expected molecular weights of VP0 (39 kDa), VP1 (37 kDa), and VP3 (29 kDa), suggesting that the P1 protein was successfully expressed and processed into VP0, VP1, and VP3 capsid proteins by 3CD. Moreover, VP0, VP1, and VP3 proteins mainly co-sedimented in fraction numbers #6 to #8 (Fig. 1b), indicating that the three subunits were capable of assembling into particles. Transmission electron microscopy of the peak fractions revealed that CVA10-VLPs were spherical in shape with diameters of ~30 nm (Fig. 1c). In addition, SDS-PAGE analysis of the purified CVA10-VLP sample showed three major protein bands that correspond to VP0, VP1, and VP3 proteins as determined by western blotting, whereas the control antigen, which was generated from uninfected Sf9 cells using the same purification protocol, did not yield any detectable bands (Fig. 1d). Altogether, these data demonstrate that CVA10-VLP can be successfully produced using the baculovirus-insect cell expression system.

### Formulation of monovalent and tetravalent VLP vaccines

**Fig. 2:**
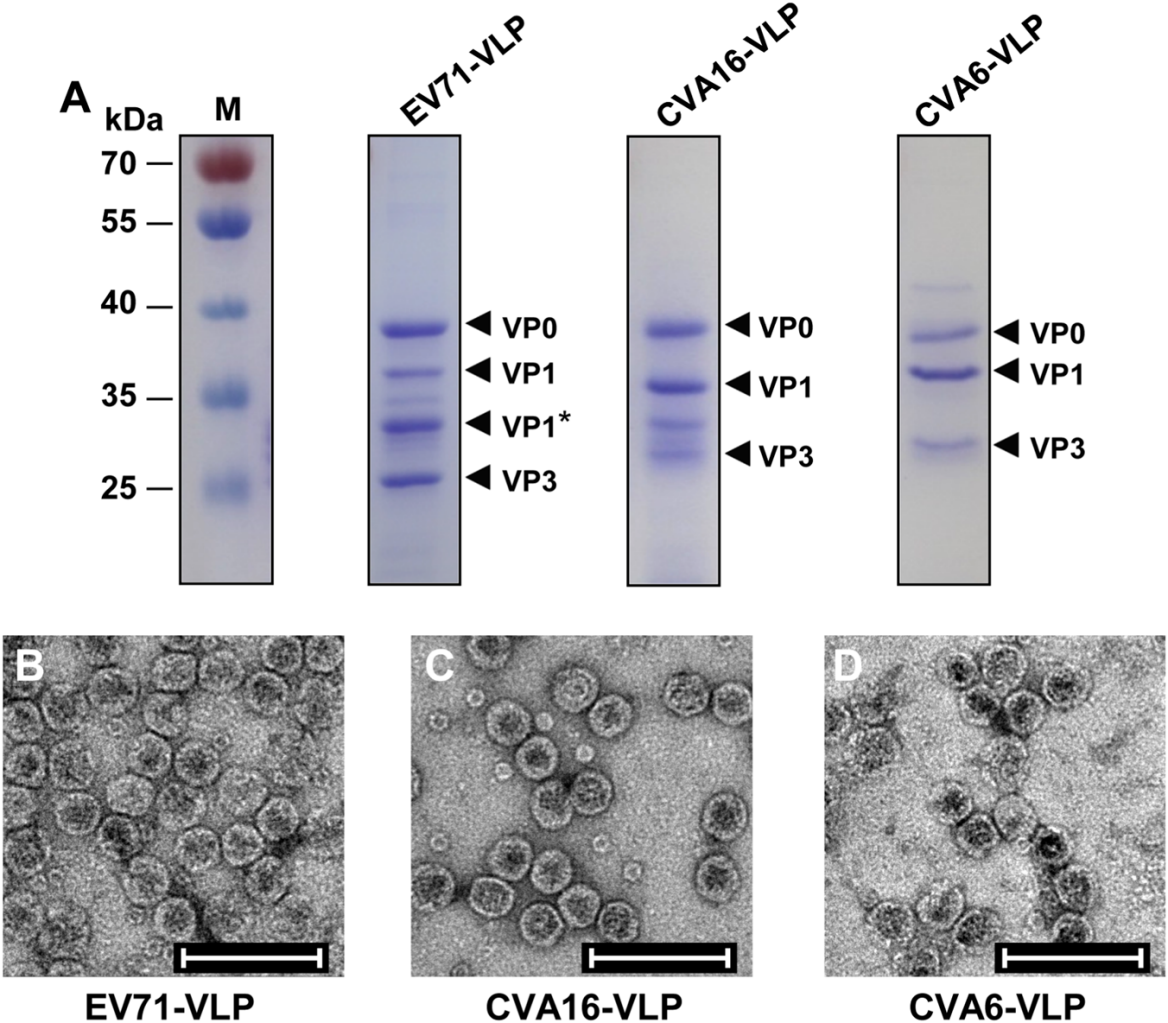
Characterization of EV71-VLP, CVA16-VLP, and CVA6-VLP. **a** SDS-PAGE analysis of purified EV71-VLP, CVA16-VLP, and CVA6-VLP. Lane M, protein marker. **b**–**d** Electron microscopy micrographs of purified **b** EV71-VLP, **c** CVA16-VLP, and **d** CVA6-VLP. Scale bar = 100 nm

Similarly, EV71-VLP, CVA16-VLP, and CVA6-VLP were generated in Sf9 insect cells infected with recombinant baculoviruses that co-expressed P1 and 3CD proteins derived from EV71, CVA16, and CVA6, respectively. The purified VLPs were then analyzed by SDS-PAGE. As shown in Figure 2a, EV71-VLP, CVA16-VLP, and CVA6-VLP showed three specific protein bands between 25 and 40 kDa, representing full-length VP0, VP1, and VP3 subunit proteins, in line with findings from previous studies^27,34^. In addition, one band (labeled VP1*), corresponding to the partially cleaved VP1 protein, was detected in the EV71-VLP sample (Fig. 2a), consistent with the results from previous characterizations of EV71-VLP^34,36^. The identities of VP0, VP1/VP1*, and VP3 of each VLP were confirmed by western blotting with subunit protein-specific polyclonal antibodies (data not shown). Electron microscopy analysis of purified VLPs showed round particles with diameters of ~30 nm (Fig. 2b-d), further demonstrating the successful formation of EV71-VLP, CVA16-VLP, and CVA6-VLP in insect cells. The size and morphology of these VLP particles were similar to those reported in previous studies^27,29,37^. A small number of small particles (~11 nm) were also observed in the EV71-VLP and CVA16-VLP samples (Fig. 2b, c), probably representing intermediate forms of VLP assembly, which have been reported in a previous study on bovine enterovirus^38^.

The four purified VLPs were mixed separately with the Alhydrogel adjuvant, yielding the four experimental monovalent VLP vaccines, each of which contained 1 μg/dose of corresponding VLP and 500 μg/dose of aluminum hydroxide. To make a tetravalent vaccine, the four types of VLPs were combined at a ratio of 1:1:1:1 and then formulated with the adjuvant. A single dose of the tetravalent VLP vaccine (designated Tetra-VLP) contained 1 μg of each VLP and 500 μg of aluminum hydroxide. For comparison, the antigen prepared from uninfected Sf9 cells was mixed with 500 μg/dose of aluminum hydroxide, serving as the control in immunization studies.

### Antibody responses elicited by immunization with VLPs

**Fig. 3:**
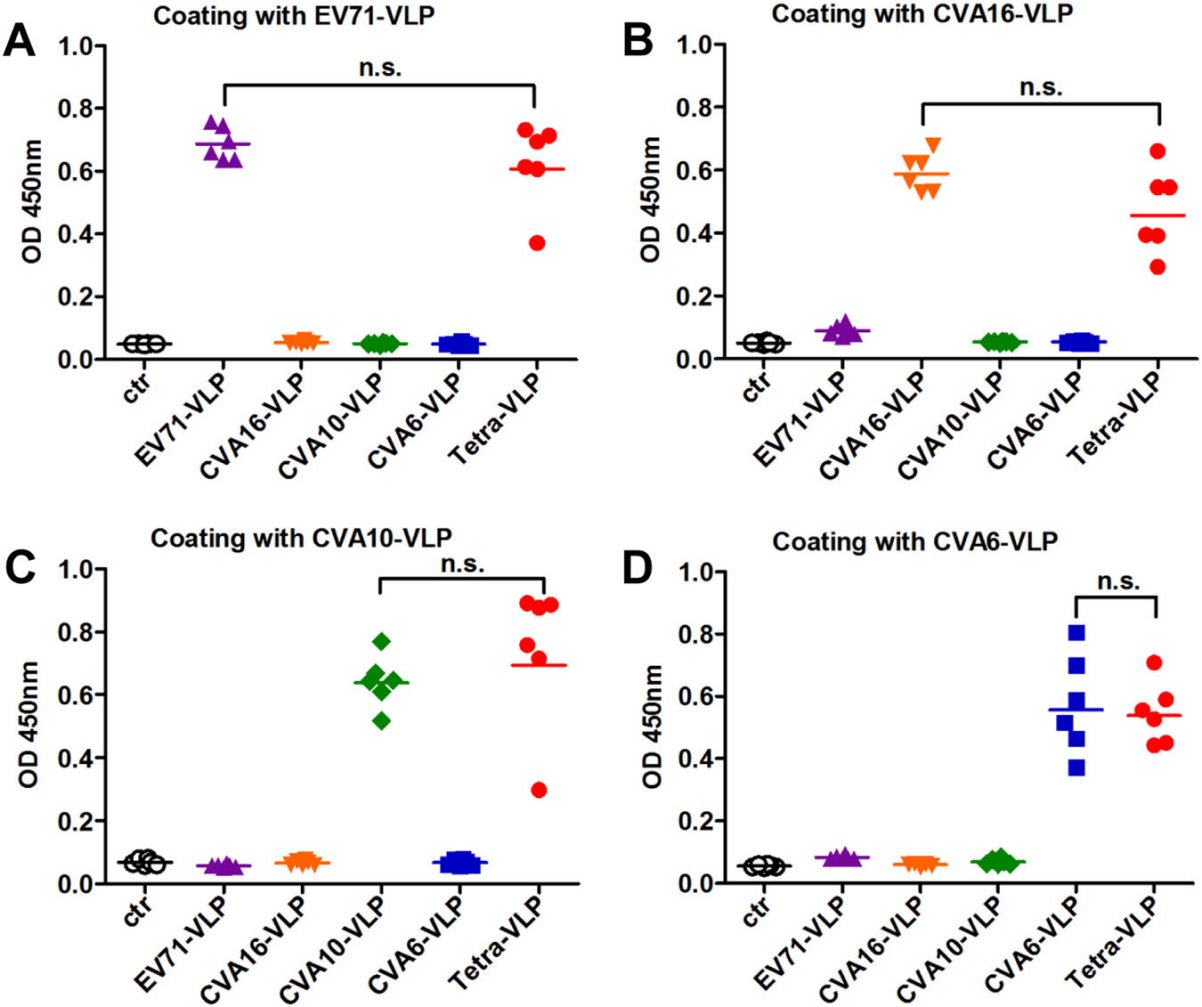
Serum antibody responses elicited by immunization with VLPs. Groups of six BALB/c mice were immunized with the control antigen (ctr), EV71-VLP, CVA16-VLP, CVA10-VLP, CVA6-VLP, or the tetravalent VLP (VLPs of EV71, CVA16, CVA10, and CVA6; termed Tetra-VLP). Serum samples were harvested from each mouse two weeks after final immunization, diluted 1:1000, and then analyzed for antigen-specific IgG antibodies by ELIZA using **a** EV71-VLP, **b** CVA16-VLP, **c** CVA10-VLP, and **d** CVA6-VLP as capture antigens. Each symbol represents a mouse, and the solid line indicates the geometric mean value of the group. Statistical significance was determined by a two-tailed Student’s *t*-test and is indicated as follows: n.s., no significant difference (*P* ≥ 0.05); *P < 0.05; **P < 0.01; and ***P < 0.001

To assess the immunogenicity of VLPs, groups of adult BALB/c mice were immunized twice intraperitoneally (i.p.) with EV71-VLP, CVA16-VLP, CVA10-VLP, CVA6-VLP, or Tetra-VLP at an interval of three weeks. For comparison, the control antigen prepared from uninfected Sf9 cells was used to inject another group of mice. Antisera were obtained two weeks after the last vaccination and then analyzed for antigen-specific IgG antibodies by ELIZA using EV71-VLP, CVA16-VLP, CVA10-VLP, or CVA6-VLP as coating antigens. As shown in Figure 3, only background levels of binding were detected for the control antigen group. The monovalent VLP-immunized mouse sera reacted with their corresponding antigens but not the other VLP antigens (Fig. 3), suggesting that antigen-specific antibody responses developed in mice following immunization with monovalent VLPs. In contrast, all sera from mice vaccinated with Tetra-VLP displayed strong reactivity to all capture antigens (Fig. 3). Furthermore, no significant difference (*P* > 0.05) in antigen-binding capacity was observed between the Tetra-VLP group and its monovalent counterparts, indicating that antibody responses elicited by monovalent and tetravalent VLP vaccines were comparable.

**Fig. 4:**
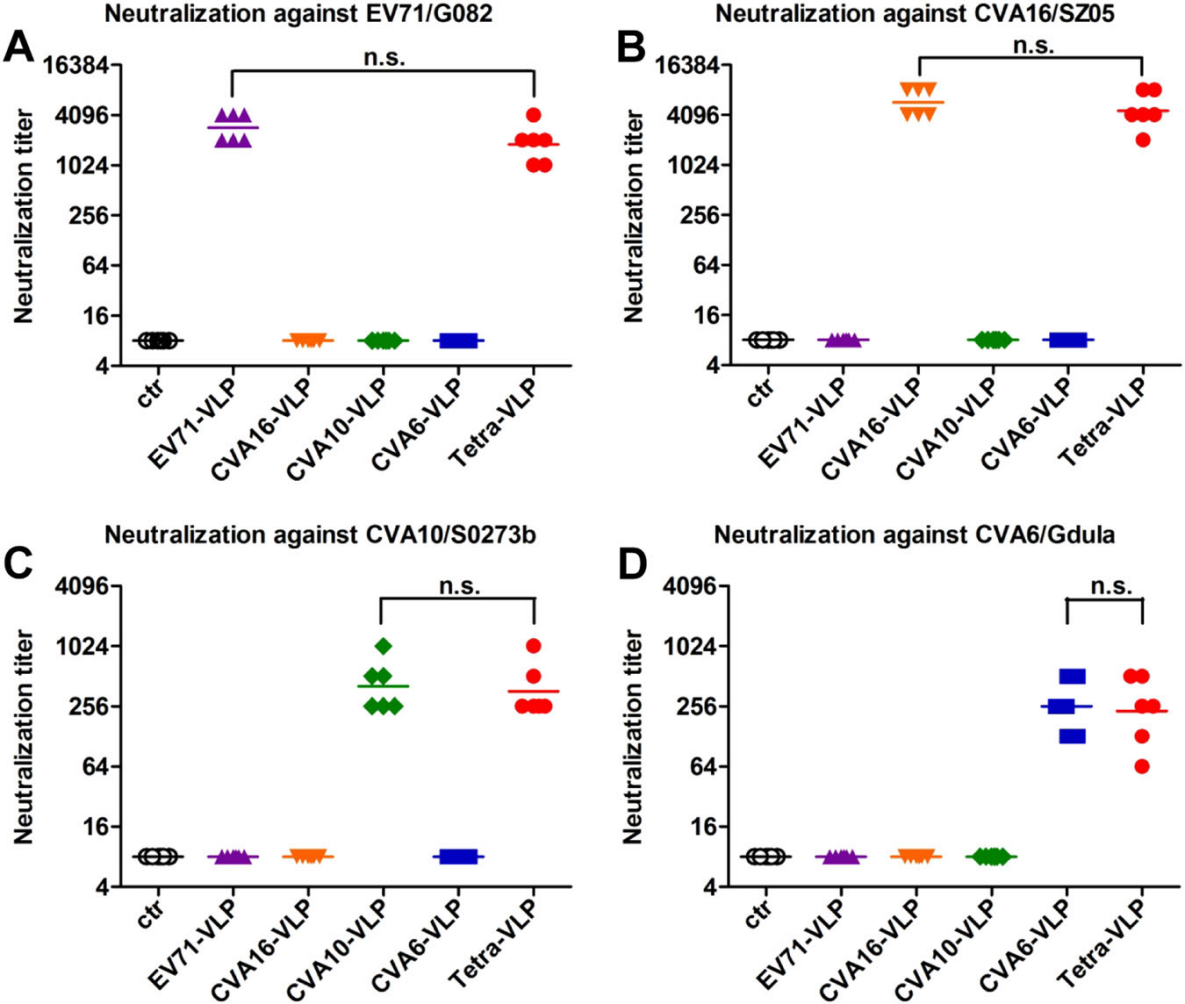
Neutralizing ability of VLP-immunized sera. Mouse antisera obtained two weeks after the last immunization dose were subjected to micro-neutralization assays to measure neutralizing antibody titers against **a** EV71/G082, **b** CVA16/SZ05, **c** CVA10/S0273b, and **d** CVA6/Gdula. For geometric mean titer (GMT) calculation, a titer of 8 was assigned to serum samples with no detectable neutralizing antibodies at the lowest dilution tested (1:16). Each symbol represents a mouse, and the solid line denotes the geometric mean value of each group. Statistical significance is indicated as follows: n.s., no significant difference (*P* ≥ 0.05); *P < 0.05; **P < 0.01; and ***P < 0.001

Individual antisera taken two weeks after the last vaccine dose were further tested for their ability to neutralize EV71, CVA16, CVA10, and CVA6 viruses in vitro using a micro-neutralization assay. As shown in Figure 4, the control antisera did not exhibit virus-neutralizing activities at the lowest dilution tested (1:16), whereas anti-EV71-VLP, anti-CVA16-VLP, anti-CVA10-VLP, and anti-CVA6-VLP sera potently neutralized their corresponding homologous viruses EV71/G082, CVA16/SZ05, CVA10/S0273b, and CVA6/Gdula with geometric mean titers (GMTs) of 2896, 5793, 406, and 256, respectively. However, all monovalent VLP-immunized sera failed to neutralize the heterologous viruses tested at the 1:16 dilution (Fig. 4). In contrast, all sera from the Tetra-VLP group strongly neutralized EV71/G082, CVA16/SZ05, CVA10/S0273b, and CVA6/Gdula with GMTs of 1825, 4598, 362, and 228, respectively (Fig. 4); this was comparable to the neutralizing antibody titers induced by their corresponding monovalent vaccines, indicating that the antigenic components of the tetravalent formulation exhibited good compatibility in neutralizing antibody induction.

To measure the breadth of neutralization, antisera obtained two weeks following the final vaccination were pooled for each group and further analyzed for their cross-neutralization activities against a panel of EV71, CVA16, CVA10, and CVA6 strains. As shown in Table 1, anti-EV71-VLP, anti-CVA16-VLP, anti-CVA10-VLP, and anti-CVA6-VLP sera could neutralize all tested strains of the respective virus but did not exhibit any significant neutralization effects on heterologous viruses. In contrast, pooled sera from the Tetra-VLP group efficiently neutralized all EV71, CVA16, CVA10, and CVA6 strains (Table 1). These results indicate that the tetravalent VLP vaccine can induce balanced and broad neutralizing antibody responses against EV71, CVA16, CVA10, and CVA6 viruses.

**Table 1:** Neutralization activity of pooled antisera against a panel of enteroviruses

Pooled antisera against	Neutralization titer against										
	EV71/G082	EV71/FY09-2	EV71/BrCr	EV71/MAV-W	CVA16/SZ05	CVA16/G08	CVA16/MAV	CVA10/S0273b	CVA10/S0148b	CVA10/Kowalik	CVA6/Gdula
Control	<32	<32	<32	<32	<32	<32	<32	<32	<32	<32	<32
EV71-VLP	4096	4096	1024	512	<32	<32	<32	<32	<32	<32	<32
CVA16-VLP	<32	<32	<32	<32	8192	8192	8192	<32	<32	<32	<32
CVA10-VLP	<32	<32	<32	<32	<32	<32	<32	256	128	64	<32
CVA6-VLP	<32	32	32	32	<32	<32	32	<32	<32	<32	512
Tetra-VLP	2048	2048	512	256	4096	4096	8192	256	64	64	512

The lowest serum dilution tested is 1:32

### Duration of VLP-elicited neutralizing antibody responses

**Fig. 5:**
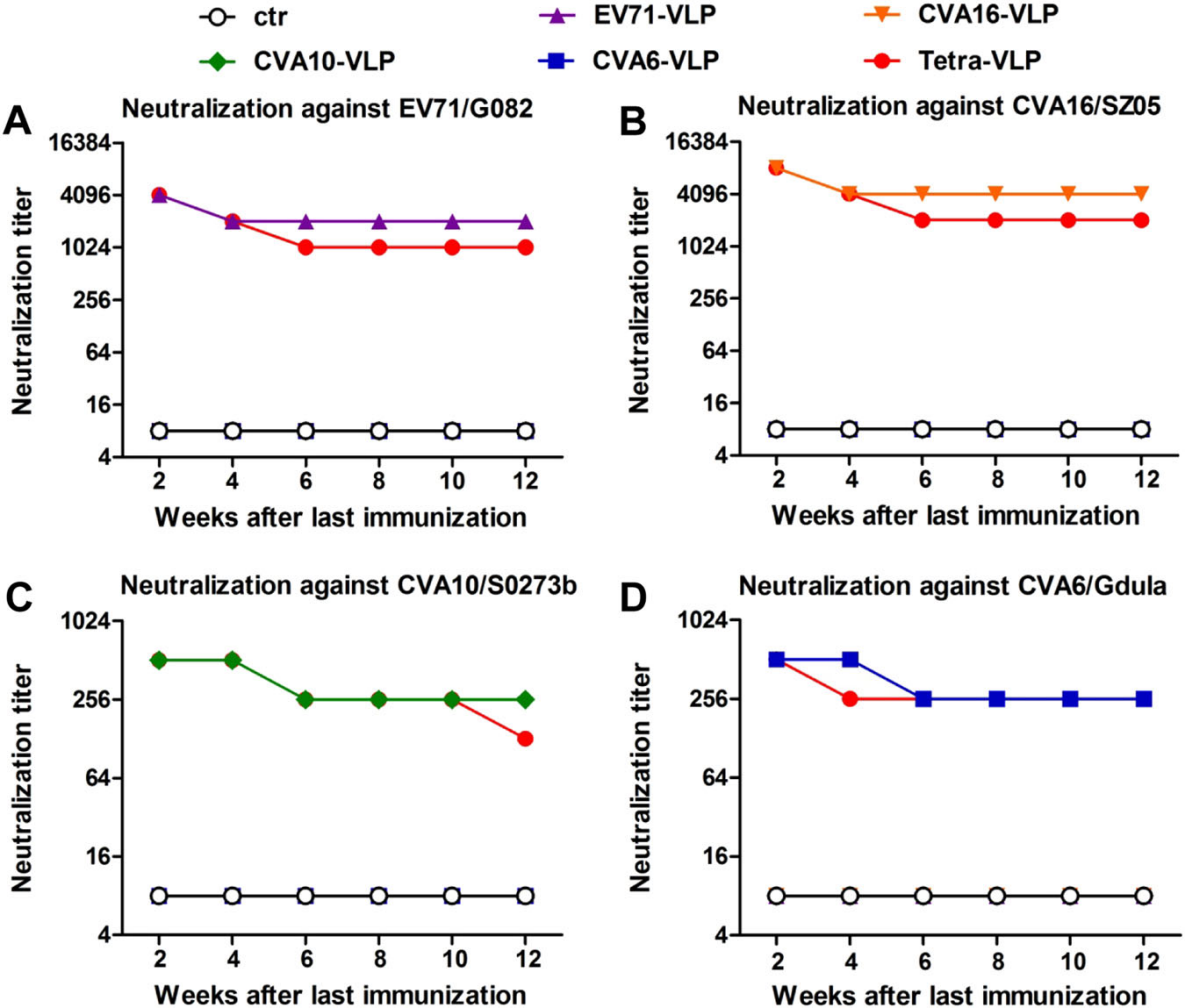
Duration of VLP-elicited neutralizing antibody responses. Antisera were taken from each immunized mouse every two weeks after the last vaccination dose and pooled for each group. The resulting antisera pools were subjected to micro-neutralization assays to detect neutralizing antibody titers against **a** EV71/G082, **b** CVA16/SZ05, **c** CVA10/S0273b, and **d** CVA6/Gdula. Serum samples that did not confer any neutralization at the starting dilution of 1:16 were assigned a titer of 8

To measure the persistence of the antibody response, serum samples from immunized mice were obtained at 2, 4, 6, 8, 10, and 12 weeks after the final immunization. Individual serum samples were pooled for each group and each time point, and the resulting antisera pools were analyzed for neutralizing antibody titers against EV71/G082, CVA16/SZ05, CVA10/S0273b, and CVA6/Gdula. Figure 5 shows that Tetra-VLP yielded similar neutralizing antibody titer profiles as its monovalent counterparts; the levels of neutralizing antibodies were highest two weeks after the last immunization and then decreased slightly over time and persisted for at least 12 weeks. These results indicate that neutralizing antibody responses induced by the tetravalent VLP vaccine were long-lasting.

### In vivo protective efficacy of VLP vaccines against lethal viral challenges

Our previous studies demonstrated that the mouse-adapted EV71 strain EV71/MAV-W, mouse-adapted CVA16 strain CVA16/MAV, CVA10 clinical isolate CVA10/S0148b, and CVA6 clinical isolate CVA6/S0087b were able to infect ICR suckling mice, resulting in limb weakness, paralysis, and death^26–28,39^. Therefore, these strains were used for challenge experiments in the present study.

**Fig. 6:**
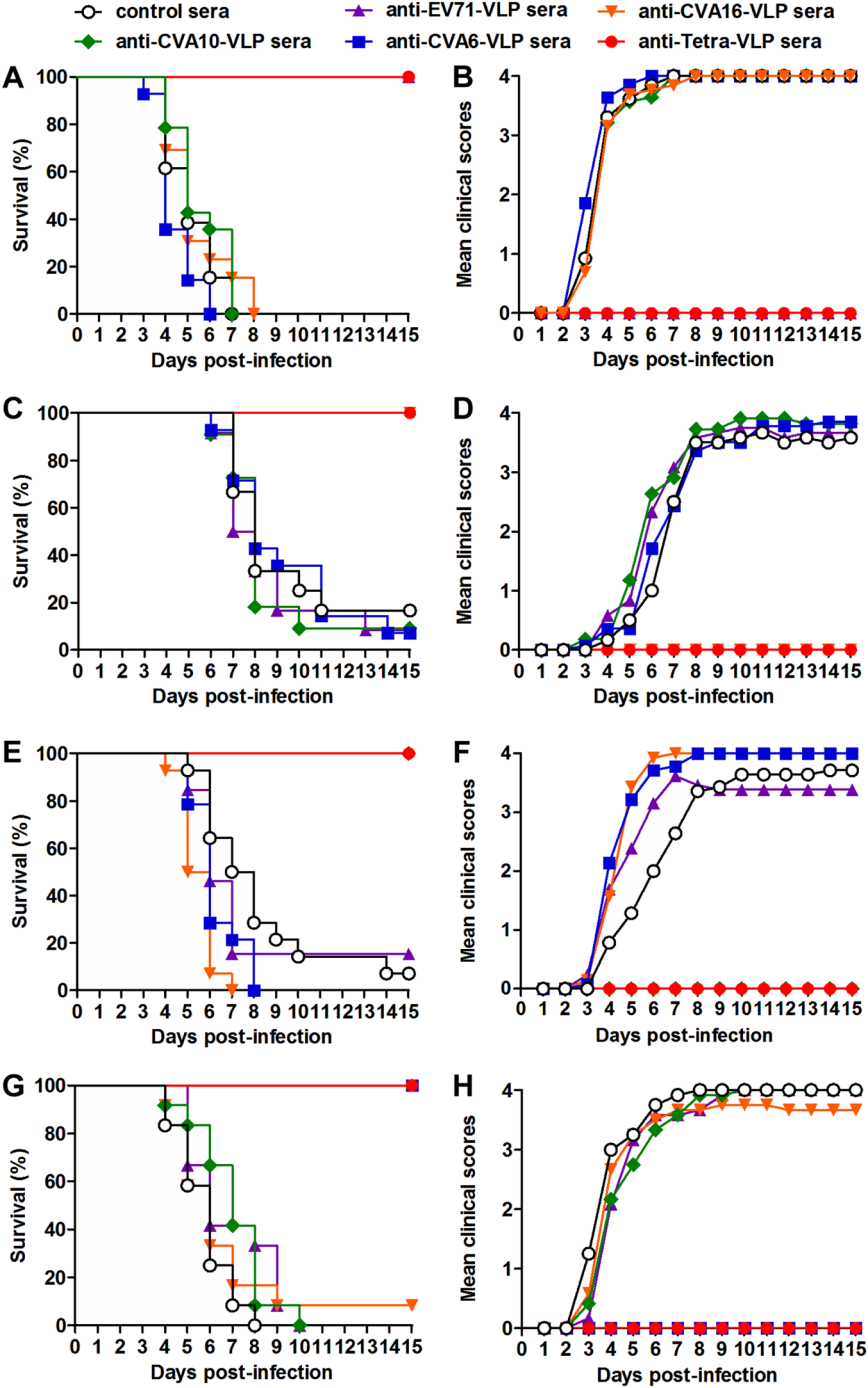
Protective efficacy of anti-VLP sera against lethal viral challenges in neonatal mice. Groups of six-day-old ICR mice (*n* = 11–14 mice/group) were injected i.p. with pooled anti-EV71-VLP, anti-CVA16-VLP, anti-CVA10-VLP, anti-CVA6-VLP, anti-Tetra-VLP, or control sera. One day later, the suckling mice were i.p. challenged with **a**, **b** EV71/MAV-W, **c**, **d** CVA16/MAV, **e**, **f** CVA10/S0148b, or **g**, **h** CVA6/S0087b. After challenge, all mice were monitored daily for (**a**, **c**, **e**, **g**) survival and (**b**, **d**, **f**, **h**) clinical score for 15 days. Clinical scores were graded as follows: 0, healthy; 1, reduced mobility; 2, limb weakness; 3, paralysis; and 4, death

To determine the protective efficacy of monovalent and tetravalent VLP vaccines, groups of naive ICR mice (six days old) were administrated i.p. with pooled anti-EV71-VLP, anti-CVA16-VLP, anti-CVA10-VLP, anti-CVA6-VLP, anti-Tetra-VLP, or control sera, followed by infection one day later with lethal doses of EV71/MAV-W, CVA16/MAV, CVA10/S0148b, or CVA6/S0087b. The suckling mice were observed daily for clinical scores and survival for 15 days. After challenges, mice treated with control antisera started to display clinical symptoms at 3–4 dpi, and 83–100% of these mice ultimately died (Fig. 6). Mice receiving monovalent VLP-immunized sera were completely protected from homotypic virus infections but exhibited symptoms and mortality similar to those in the control group when infected with heterotypic viruses. Treatment with the Tetra-VLP immune sera fully protected the recipient mice from single infections with EV71, CVA16, CVA10, or CVA6 (Fig. 6).

**Fig. 7:**
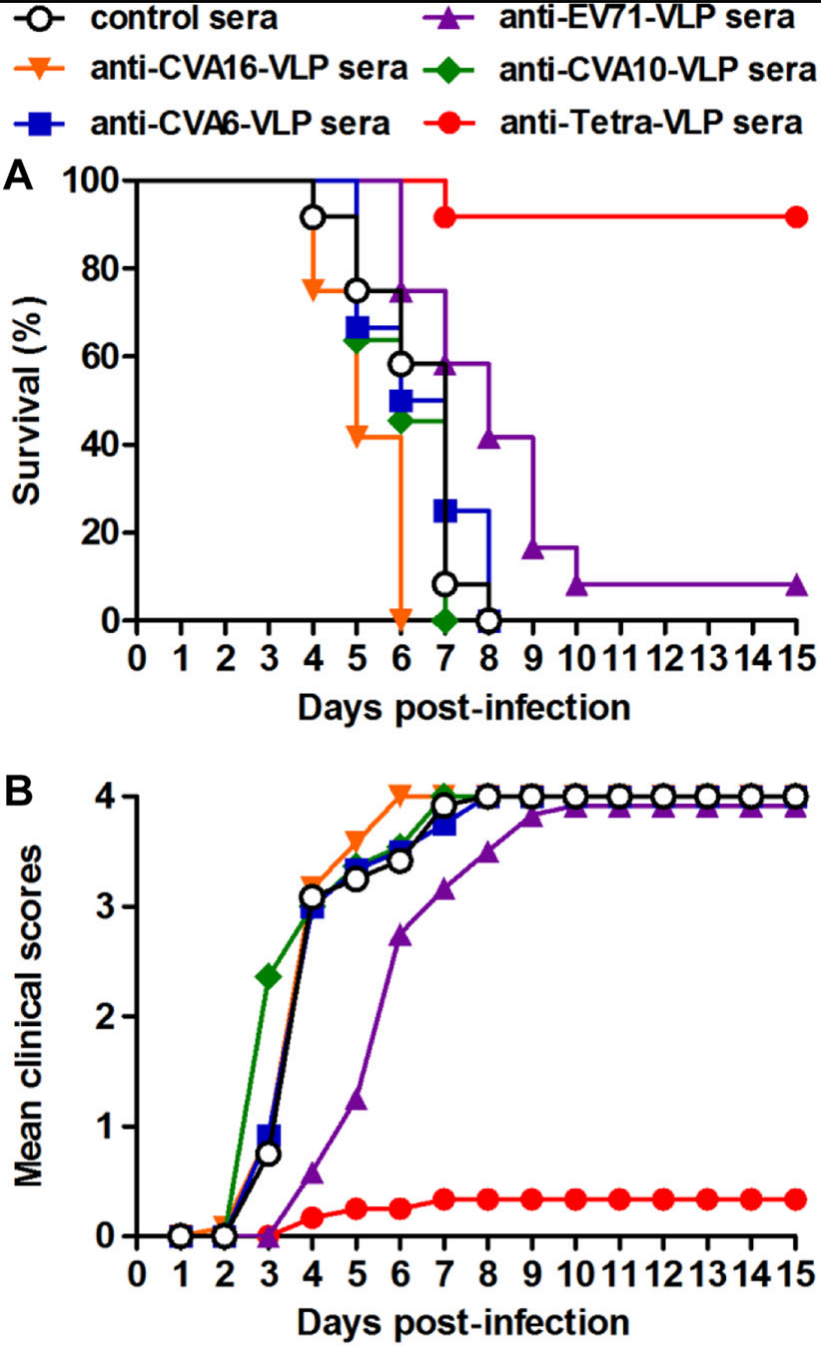
Anti-Tetra-VLP sera effectively protected recipient suckling mice against lethal viral co-infection. Groups of six-day-old ICR mice (*n* = 11–12/group) were i.p. administered with pooled anti-EV71-VLP, anti-CVA16-VLP, anti-CVA10-VLP, anti-CVA6-VLP, anti-Tetra-VLP, or control sera, followed by simultaneous inoculation one day later with EV71/MAV-W, CVA16/MAV, CVA10/S0148b, and CVA6/S0087b. **a** Survival and **b** clinical score were monitored daily for 15 days following challenge. Clinical scores were graded as described in the legend of Figure 5

During HFMD epidemics, co-infections with two or more of the EV71, CVA16, CVA6, and CVA10 viruses frequently occurred^23^. To evaluate the protective potential of our vaccine candidates against co-infection, we developed an in vivo co-infection model in which neonatal mice were challenged with a mixture of the EV71/MAV-W, CVA16/MAV, CVA10/S0148b, and CVA6/S0087b strains. As a control, mice that were administered the control antisera and one day later inoculated with the virus mixture gradually developed symptoms and eventually died within 8 dpi (Fig. 7), and the disease symptoms and mortality rate of these co-infected mice were comparable to those observed in the mice solely inoculated with EV71 or CVA6, but were more severe than those displayed in mice solely inoculated with CVA16 and CVA10 (Fig. 6). As shown in Figure 7, following co-infection, the mice receiving anti-Tetra-VLP sera were well protected with a survival rate of 92%, whereas all or most of the mice in the four anti-monovalent-VLP treatment groups exhibited disease symptoms and then died (Fig. 7). Overall, the above challenge results indicate that the monovalent VLP vaccine can only protect against homotypic virus infection, while the tetravalent VLP vaccine is able to offer efficient protection against single or mixed infections with EV71, CVA16, CVA10, and CVA6 viruses.

## Discussion

Development of safe and efficacious HFMD vaccines is challenging because HFMD can be caused by multiple enterovirus serotypes with no cross-protection among them^24^. For example, licensed monovalent EV71 vaccines cannot protect against other etiologic agents of HFMD^24^. Efforts have been made toward developing multivalent vaccines for broader protection against HFMD. Specifically, Caine et al. reported that a trivalent vaccine candidate containing inactivated EV71, CVA16, and CVA6 protected mice against challenge with each of the three viruses^40^. In a separate study, an inactivated tetravalent vaccine candidate was found to induce, in mice and rabbits, serum antibodies capable of neutralizing EV71, CVA16, CVA10, and CVA6 in vitro;^41^ however, its in vivo protective efficacy was not assessed. Here we report, to our knowledge, the first development of a VLP-based tetravalent HFMD vaccine targeting EV71, CVA16, CVA10, and CVA6.

Immunologic interference is an issue that is often encountered in formulating multivalent combination vaccines and has been documented in previous studies on tetravalent dengue and trivalent poliovirus vaccines^42,43^. Our present study shows that the tetravalent VLP vaccine can induce broadly neutralizing antibodies against EV71, CVA16, CVA10, and CVA6, whereas the monovalent vaccine-elicited antisera can only neutralize the homotypic viruses (Fig. 4 and Table 1). In addition, the antisera’s neutralizing capacities against a specific virus were not significantly different between the tetravalent vaccine group and the corresponding monovalent vaccine group (Fig. 4), indicating a good compatibility among the four VLP components regarding their immunogenicity. These results should encourage further development of the tetravalent VLP vaccine. We should mention that the CVA10-neutralizing and CVA6-neutralizing titers for the monovalent CVA10-VLP (GMT = 406) and the monovalent CVA6-VLP (GMT = 256) groups, respectively, were in general lower than the EV71-neutralizing and CVA16-neutralizing titers induced by the corresponding monovalent EV71-VLP (GMT = 2896) and monovalent CVA16-VLP (GMT = 5793) vaccine (Fig. 4). A similar observation has been made in a previous study^41^. Specifically, Liu et al. reported that an inactivated tetravalent EV71/CVA16/CVA10/CVA6 vaccine induced neutralizing antibodies against all four viruses with titers of 708 for EV71, 22 for CVA16, 16 for CVA10, and 100 for CVA6 in mice^41^. These observations suggest that antigens (either VLP or inactivated virus) derived from CVA10, CVA6, and perhaps CVA16 as well, may be less immunogenic than those from EV71. It is possible that strong T-cell epitopes might solely exist in EV71 antigens, leading to the observed potent immunogenicity of EV71 antigens. The exact mechanisms underlying the drastic difference in immunogenicity between EV71 and other viruses (e.g., CVA10) remain to be elucidated.

Our study reveals that the tetravalent VLP vaccine can elicit broadly neutralizing antibodies against EV71, CVA16, CVA10, and CVA6 and therefore represents a promising and broadly effective HFMD vaccine candidate. To advance this vaccine candidate into the next development stage, its immunogenicity needs to be further characterized. For example, as a logical next step, a more detailed analysis of the immunogenicity of the tetravalent VLP vaccine in mice, such as the effects of different doses and different vaccination strategies, should be performed. For any given vaccine, its immunogenicity in non-human primates may not be the same as in mice. In particular, in a side-by-side comparison, EV71-VLP was found to elicit lower neutralizing antibody titers in macaque monkeys than did inactivated EV71^44^, whereas compared with the inactivated EV71 vaccine, EV71-VLP was more potent at inducing neutralizing antibodies and conferred better protection in mice^45^. Therefore, it is essential that our tetravalent vaccine candidate be further evaluated in non-human primates.

In the present study, we performed antisera transfer/virus challenge experiments to determine the protective efficacy of the tetravalent VLP vaccine in neonatal mouse models. Our results showed that antisera from the tetravalent VLP vaccine-immunized mice could confer complete protection against lethal infection with any one of the four viruses, whereas antisera from the monovalent VLP groups could protect against homotypic but not heterotypic virus infections (Fig. 6). Moreover, the tetravalent VLP immune sera potently protected mice from lethal co-infection with all four viruses, while the monovalent-VLP antisera conferred no or minimal protection against co-infection (Fig. 7). The latter finding is particularly significant, as co-circulation of EV71, CVA16, CVA6, and/or CVA10 during HFMD epidemics leads to an increased incidence of co-infections that have been associated with disease severity in patients^23^.

In summary, our study demonstrates that the VLP-based tetravalent vaccine can efficiently induce a broad-spectrum, balanced, and durable neutralizing antibody response and can protect against the most common HFMD pathogens, thus representing a promising broadly effective HFMD vaccine candidate worthy of further development.

## Materials and methods

### Cells and viruses

Human rhabdomyosarcoma cells (ATCC, CCL-136) were cultured as described previously^46^. *Spodoptera frugiperda* Sf9 insect cells were cultured at 27 °C in Sf-900 II SFM (serum-free medium) (Invitrogen, USA). EV71 strains used in the present study include the prototype strain EV71/BrCr, mouse-adapted strain EV71/MAV-W, and clinical strains EV71/G082 and EV71/FY09-2^34,39^. CVA16 clinical strains CVA16/SZ05 and CVA16/G08 and a mouse-adapted CVA16 strain CVA16/MAV were described in a previous study^26^. CVA10 prototype strain CVA10/Kowalik and two CVA10 clinical isolates, CVA10/S0148b and CVA10/S0273b, were described in a previous study^28^. CVA6 clinical isolate CVA6/S0087b and prototype strain CVA6/Gdula have also been described previously^47^. CVA6/Gdula virus stock was prepared as previously described^40^. The 50% tissue culture infectious dose (TCID_50_) for the EV71, CVA16, CVA10, and CVA6 viruses was determined according to the Reed–Muench method^48^. CVA6/S0087b was quantified by real-time reverse transcription PCR to determine the absolute viral genome copy number as described previously^27^.

### Antibodies

Polyclonal antibodies against VP0, VP1, and VP3 proteins of CVA10 were described previously^28^.

### Vector construction

To construct recombinant baculovirus vectors for EV71-VLP expression, the P1 gene of EV71/G082 was codon-optimized, synthesized, and then inserted into the pFastBac™ Dual vector (pFBD; Invitrogen) under the PH promoter, yielding plasmid pFBD-EV71-P1. The 3CD gene fragment of EV71/G082 was then cloned into pFBD-EV71-P1 under the control of the p10 promoter, to construct pFBD-EV71-P1/3CD. Similarly, the optimized P1 gene of CVA10/S0273b and the 3CD gene of CVA10/Kowalik were separately cloned into the same backbone vector pFBD under the PH and p10 promoters, respectively, resulting in plasmid pFBD-CVA10-P1/3CD. The optimized P1 gene of CVA6/SZc173/13 (GenBank ID: KF682362) and the 3CD gene of CVA6/Gdula were separately cloned into the same pFBD plasmid under the control of the PH and p10 promoters, respectively, to generate pFBD-CVA6-P1/3CD. Construction of the recombinant plasmid pFBD-CVA16-P1/3CD for CVA16-VLP expression has been described previously^29^.

### Generation of recombinant baculoviruses

The plasmids pFBD-EV71-P1/3CD, pFBD-CVA10-P1/3CD, and pFBD-CVA6-P1/3CD were separately transformed into competent *Escherichia. coli* DH10Bac cells (Invitrogen) for generating recombinant bacmids. The resultant bacmid DNA was separately transfected into Sf9 insect cells to obtain the corresponding recombinant baculoviruses designated Bac-EV71-P1/3CD, Bac-CVA10-P1/3CD, and Bac-CVA6-P1/3CD using the Bac-to-Bac baculovirus expression system (Invitrogen) according to the manufacturer’s instructions. Generation of the recombinant baculovirus Bac-CVA16-P1/3CD for CVA16-VLP expression has been described previously^29^.

### Preparation of VLPs and the control antigen

To generate VLPs, suspension cultures of Sf9 (2 × 10^6^ cells/mL) were infected with the recombinant baculoviruses Bac-EV71-P1/3CD, Bac-CVA16-P1/3CD, Bac-CVA10-P1/3CD, or Bac-CVA6-P1/3CD at a multiplicity of infection of 1 followed by culturing at 27 °C for 3 days. Sf9 cells from each culture were then collected by centrifugation and lysed with 0.15 M PBS containing 1% NP-40. Cell lysates were centrifuged at 12,000 rpm for 15 min to remove cellular debris, and the resultant supernatants were precipitated overnight at 4 °C with 8% (w/v) polyethylene glycol 8000 and 200 mM NaCl. After centrifugation at 12,000 rpm for 15 min, the resulting pellets were collected and resuspended in 0.15 M PBS buffer, followed by clarification by centrifugation. Next, 20% sucrose cushion and 10–50% sucrose-gradient ultracentrifugation steps were carried out as previously described^37^. Finally, VLP-rich fractions were pooled and buffer-exchanged into 0.15 M PBS buffer using Amicon Ultra 100 K centrifugal filters (Millipore, USA). For comparison, the control antigen was generated from uninfected Sf9 cells following the same protocol. Purified VLPs and control antigen were quantified using the Bradford protein assay kit (Bio-Rad, USA) according to the manufacturer’s instructions.

### SDS-PAGE and western blotting

SDS-PAGE and western blotting analyses of purified VLPs or gradient fractions were performed as described previously^49^ but with a minor modification: polyclonal antibodies against VP0, VP1, or VP3 proteins of CVA10 served as the primary antibodies.

### Electron microscopy

Purified VLP samples were separately adsorbed on carbon-coated copper grids, negatively stained with 0.5% aqueous uranyl acetate, and then imaged with a Tecnai G2 Spirit transmission electron microscope (FEI, USA) at 120 kV.

### Mouse immunization

All animal study protocols were approved by the Institutional Animal Care and Use Committee at the Institut Pasteur of Shanghai. All mice were obtained from Shanghai Laboratory Animal Center (SLAC, China).

Purified EV71-VLP, CVA16-VLP, CVA10-VLP, and CVA6-VLP (1 μg/dose) were mixed separately with Alhydrogel^®^ adjuvant (500 μg/dose; Invivogen, USA) by vortexing to produce monovalent VLP vaccines. Similarly, VLPs of EV71, CVA16, CVA10, and CVA6 (1 μg of each antigen/dose) and the adjuvant (500 μg/dose) were thoroughly mixed, resulting in the tetravalent VLP (termed Tetra-VLP) vaccine. The control antigen was formulated with the adjuvant and used as a control. Groups of six female BALB/c mice, 6–8 weeks old, were administrated i.p. with the experimental vaccines at weeks 0 and 3. Blood samples were harvested from each mouse at weeks 2, 4, 6, 8, 10, and 12 after the final immunizations and then heat-inactivated at 56°C for 30 min to destroy complement.

### Serum antibody measurement and neutralization assay

Antigen-specific IgG antibodies in mouse sera were measured by indirect ELIZA. Briefly, 96-well ELIZA plates (Nunc, USA) were coated overnight at 4 °C with 50 ng/well of EV71-VLP, CVA16-VLP, CVA10-VLP, or CVA6-VLP, followed by blocking in 5% milk in PBS-Tween20 (PBST). Serum samples collected two weeks after the last immunizations were added at a dilution of 1:1000 (50 μL/well) and incubated for 2 h at 37 °C. The plates were then incubated with horseradish peroxidase-conjugated anti-mouse IgG (Sigma-Aldrich, USA). Plates were washed three times with PBST between each step. TMB substrate (New Cell & Molecular Biotech, China) was added for color development, and then the absorbance at 450 nm was determined.

The neutralization titers of antisera against EV71, CVA16, CVA10, and CVA6 were determined by micro-neutralization assays as described previously^28,29,37,40^. Neutralization titers are defined as the highest serum dilutions at which no cytopathic effects are observed.

### In vivo protection assays

The protective efficacy of VLP vaccines was determined using passive immunization/challenge assays. Groups of six-day-old ICR mice were injected i.p. with 50 µL of pooled antisera from the control, EV71-VLP, CVA16-VLP, CVA10-VLP, CVA6-VLP, or Tetra-VLP groups. One day later, the suckling mice were i.p. inoculated with 1.75 × 10^5^ TCID_50_ of EV71/MAV-W, 1.0 × 10^4^ TCID_50_ of CVA16/MAV, 1.78 × 10^6^ TCID_50_ of CVA10/S0148b, 4.75 × 10^4^ copies of CVA6/S0087b, or a mixture of all four viruses (the dose of each virus in the mixture was the same as when administered individually). After viral challenge, all mice were checked daily for survival and assigned clinical scores for a period of 15 days. Clinical scores were graded as follows: 0, healthy; 1, reduced mobility; 2, limb weakness; 3, paralysis; and 4, death.

### Statistical analysis

All statistical analyses were performed using GraphPad Prism version 5. Virus-specific antibody responses and neutralizing titers were analyzed by a two-tailed Student’s *t*-test.
